# Ex Vivo Organ Cultures as Models to Study Bone Biology

**DOI:** 10.1002/jbm4.10345

**Published:** 2020-02-14

**Authors:** Teresita Bellido, Jesus Delgado‐Calle

**Affiliations:** ^1^ Department of Anatomy, Cell Biology & Physiology Indiana University School of Medicine Indianapolis IN USA; ^2^ Division of Endocrinology, Department of Medicine Indiana University School of Medicine Indianapolis IN USA; ^3^ Indiana Center for Musculoskeletal Health Indiana University School of Medicine Indianapolis IN USA; ^4^ Richard L. Roudebush Veterans Affairs Medical Center Indianapolis IN USA; ^5^ Division of Hematology/Oncology, Department of Medicine Indiana University School of Medicine Indianapolis IN USA

**Keywords:** BONE, CANCER, EX VIVO, FAT, MUSCLE, OSTEOCYTES

## Abstract

The integrity of the skeleton is maintained by the coordinated and balanced activities of the bone cells. Osteoclasts resorb bone, osteoblasts form bone, and osteocytes orchestrate the activities of osteoclasts and osteoblasts. A variety of in vitro approaches has been used in an attempt to reproduce the complex in vivo interactions among bone cells under physiological as well as pathological conditions and to test new therapies. Most cell culture systems lack the proper extracellular matrix, cellular diversity, and native spatial distribution of the components of the bone microenvironment. In contrast, ex vivo cultures of fragments of intact bone preserve key cell–cell and cell–matrix interactions and allow the study of bone cells in their natural 3D environment. Further, bone organ cultures predict the in vivo responses to genetic and pharmacologic interventions saving precious time and resources. Moreover, organ cultures using human bone reproduce human conditions and are a useful tool to test patient responses to therapeutic agents. Thus, these ex vivo approaches provide a platform to perform research in bone physiology and pathophysiology. In this review, we describe protocols optimized in our laboratories to establish ex vivo bone organ cultures and provide technical hints and suggestions. In addition, we present examples on how this technical approach can be employed to study osteocyte biology, drug responses in bone, cancer‐induced bone disease, and cross‐talk between bone and other organs © 2020 The Authors. *JBMR Plus* published by Wiley Periodicals, Inc. on behalf of American Society for Bone and Mineral Research.

## Osteocytes: Challenges and Opportunities to Study Their Role in Bone Biology

Initial calculations in human bones revealed that osteocytes are by far the most abundant bone cells, comprising more than 90% of all cells in bone, in comparison to osteoblasts (about 5%) and osteoclasts (1% to 2%).^1^, ^2^, ^3^ In addition, osteocytes inhabit permanently the entire bone volume, in contrast to osteoblasts and osteoclasts that only occupy areas of bone surfaces that require replacement for new bone. Although initially described as inactive quiescent cells buried in the mineralized tissue, osteocytes are essential for the accomplishment of the functions of the skeleton. Osteocytes coordinate bone acquisition during growth, control the maintenance of a healthy skeleton suit for locomotion, and provide protection of essential organs.^4^, ^5^, ^6^ Osteocytes orchestrate the work of osteoblasts that form bone and osteoclasts that resorb it by producing and secreting factors in response to environmental cues, thus adapting the skeleton to mechanical needs and hormonal changes. Osteocytes also contribute to the endocrine functions of bone by secreting hormones that affect other tissues; they also contribute to mineral homeostasis and hematopoiesis, which are essential for organismal life.

A small proportion (<20%) of osteoblasts on bone surfaces become surrounded by the matrix proteins that they produce, and differentiate into osteocytes.^7^, ^8^ Osteoblast–osteocyte differentiation is characterized by alterations in gene expression and is accompanied by morphological and functional cellular changes.^4^, ^5^, ^6^ During osteocytogenesis, the cuboidal shape of the highly secretory osteoblasts progressively changes to a star‐like morphology that characterizes the osteocytes. The long cytoplasmic processes exhibited by osteocytes run through canaliculi, dig in mineralized bone, and touch neighboring osteocytes, bone surface cells, and endothelial cells of the blood vessels. This extensive lacunar–canalicular system is maintained by the osteocytes' own ability to remodel their surrounding space, and it is responsible for distributing molecules secreted by osteocytes among all cells of the bone–bone marrow microenvironment and into the systemic circulation.

Reproducing the complexity of the interactions among bone cells within the osteocytic network remains a major challenge for bone biologists. Although osteocytic cell lines of rodent or human origin have been developed,^9^, ^10^, ^11^, ^12^, ^13^ they do not exhibit all features of authentic osteocytes. In addition, isolation of primary osteocytes from bone requires extensive digestions of extracellular matrix proteins, and separation from other bone and endothelial cells. This process and the subsequent in vitro culture deprive osteocytes from their native spatial surroundings and lead to changes in the cellular phenotype and morphology, thus rendering cells poorly representative of authentic osteocytes. In addition, once placed in culture, authentic osteocytes frequently lose expression of osteocytic genes and de‐differentiate back to an osteoblastic phenotype. Attempts to culture osteocytes (either cell lines or primary cells) in 3D matrices have been only partially successful because of the lack of reproducible results. Similarly, in vitro osteocyte formation from osteoblast precursor cells derived from the bone marrow, or from differentiated bones, lead only to mixed populations of osteocytes, osteoblasts, and less‐differentiated cells, limiting its use for most purposes.

**Figure 1:**
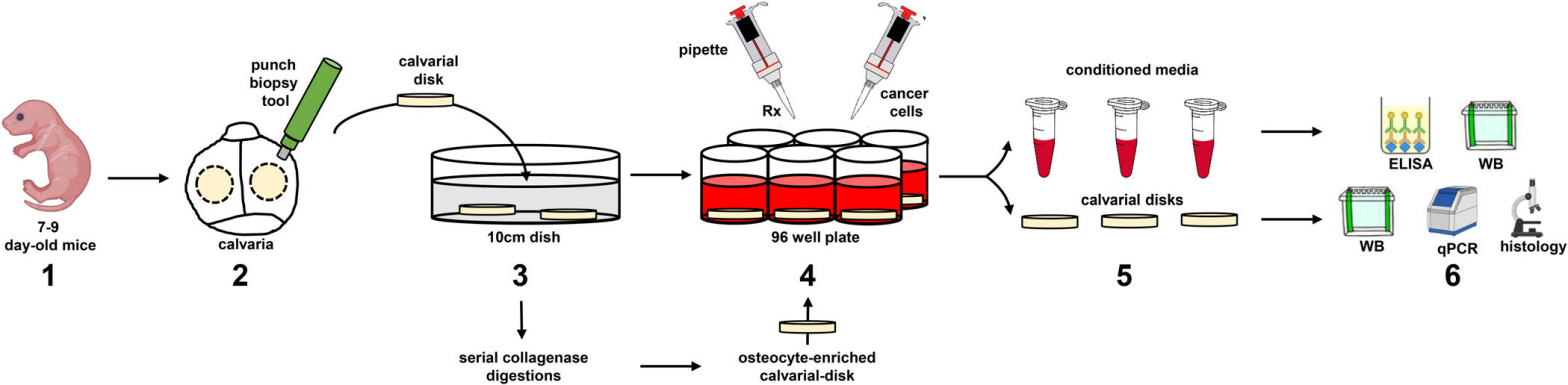
Step‐by‐step graphical depiction of the procedures described in the Practical Considerations in the Establishment of ex vivo Bone Organ Cultures section. 1. Obtain 7‐ to 9‐day‐old mice. 2. Isolate calvarial disks using a punch biopsy tool. 3. Wash the calvarial cells with PBS. If using osteocyte‐enriched bone organ cultures, perform serial collagenase digestions, wash with PBS, and incubate with complete media overnight. 4. Transfer calvarial disks to independent wells and start treatments (Rx) or add cancer cells to establish cancer–bone organ cultures. 7. At the end of the study, (a) collect conditioned media in Eppendorf tubes and either freeze or perform protein analysis by ELISA and/or Western blot (WB); and (b) collect calvarial disks and either freeze or process for protein (WB), RNA expression (qPCR), or histology.

Ex vivo bone organ cultures are a convenient alternative that bypasses the limitations of in vitro cultures of isolated osteocytes. Cultured bone explants maintain the natural position of osteocytes within the extracellular mineralized matrix, thus preserving the in vivo 3D distribution. In addition, in bone organ cultures, the innate proportion of osteocytes compared with other cells (osteoblasts, osteoclasts, bone marrow cells, and endothelial cells) is maintained. Moreover, ex vivo bone organ cultures allow the study of the effects of both genetic and pharmacologic manipulations on bone cells. In comparison to in vivo work with animal models, ex vivo organ cultures represent much less costly screening models. Importantly, findings using ex vivo bone organ cultures faithfully reproduce results seen in in vivo animal models. Ex vivo bone organ cultures were originally used for the investigation of the mechanisms underlying bone growth, bone and cartilage matrix turnover, and the effects of cancer in bone.^14^, ^15^, ^16^, ^17^, ^18^, ^19^, ^20^ More recently, this system has been employed to study mechanical loading, interactions between different bone cell types, and the molecular steps involved in bone resorption and formation. In this article, we describe the procedures optimized by our group to establish ex vivo whole‐bone organ cultures and osteocyte‐enriched bone organ cultures (Fig. 1).

## Practical Considerations in the Establishment of ex vivo Bone Organ Cultures

### Ex vivo whole‐bone organ cultures

**Figure 2:**
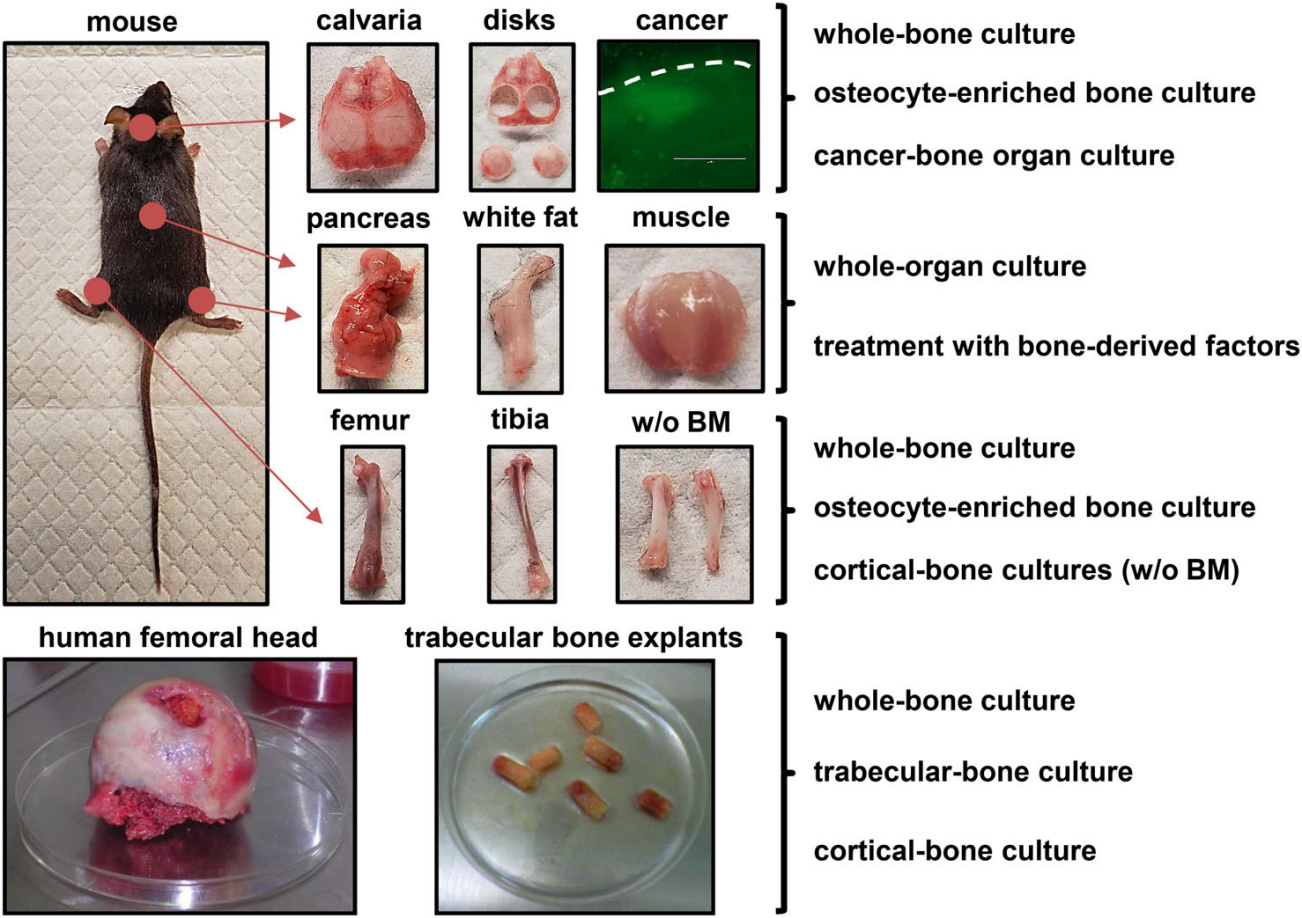
Ex vivo organ cultures with mouse and human tissues. Ex vivo organ cultures preserve key cell–cell and cell–matrix interactions and allow the study of cells in their natural niche and thus reproduce and predict in vivo responses in a time‐ and cost‐efficient manner. Multiple organ cultures can be established with tissues from the mouse. Calvarias and long bones can be used to establish different types of ex vivo bone organ cultures, including whole‐bone organ cultures, osteocyte‐enriched organ cultures, and cancer–bone organ cultures. The effects of bone‐derived factors on other organ such as muscle, fat, and pancreas can also be examined using whole‐organ cultures treated with bone‐derived factors. Lastly, ex vivo bone organ cultures established with bone explants obtained from femoral heads collected after arthroplasty can be convenient to examine responses in human bone.

Ex vivo bone organ cultures offer a unique approach to study interactions among cells in the local bone microenvironment in a controlled experimental setting where the cellular diversity of the bone and spatial distribution of cells are maintained. In Fig. 1 and Table 1, we provide a step‐by‐step guide and tips to establish whole‐bone organ cultures using both calvarias and long bones. When using calvarial bones, dip the mice in 95% ethanol three times under the hood, cut off the head with scissors, and dissect out the calvarial bone as follows: first, cut along the occipital suture line across the back of the calvaria, then along the suture line on one side toward the eye, then from pin to eye and from pin to the opposite eye. Gently, lift the calvarial bone, clean off brain and soft tissue if necessary, and place it into a dish with PBS (Phosphate‐buffered saline). After all animals are processed, punch two holes in each calvaria using a 5‐mm biopsy punch and initiate cultures as indicated in Table 1 (Fig. 2). When using long bones, dissect the tibias and femurs and place them in 24‐well plates. Remove soft tissue and periosteum via scraping and extensive washing, and initiate culture as indicated in Table 1.

**Table 1:** Step‐by‐Step Protocol to Establish ex vivo Bone Organ Cultures From Neonatal Calvarial Bones

Steps		Tips
1.	Dissect the calvarial bones (or long bones), clean muscle and blood, and transfer to a 24‐well plate filled with sterile cold PBS.	Ideally, bone organ cultures are established with calvarial disks obtained from 7‐ to 9‐day‐old mice.
2.	Punch 2 holes in each calvaria using a 5‐mm biopsy punch, avoiding cranial sutures. If using bones, cut epiphysis to allow flow of culture media into the bone.	This step normalizes the amount of bone in each well/treatment condition, thus allowing the study of protein levels in conditioned media.
3.	Transfer 5‐mm calvaria disks to a 96‐well plate containing 200 μL of media per well.	Use αMEM media supplemented with 10% FBS and 1% P/S.
4.	Collect conditioned media for assessing secreted protein levels and calvarial disks for mRNA expression.	Culture disks for up to 72 hours to achieve detectable levels of secreted factors in the media.

This protocol can be used with bones isolated from WT and genetically modified mice. When testing treatments in ex vivo bone organ cultures, we recommend performing dose‐ and time‐course experiments to determine the ideal conditions for your genes/proteins of interest.

αMEM = Minimum essential medium; P/S = Penicillin/Streptomycin; FBS = Fetal Bovine Serum.

### Ex vivo osteocyte‐enriched bone organ cultures

To enhance our ability to study osteocyte biology, our research group developed two different protocols to establish osteocyte‐enriched bone organ cultures from calvarial bones (Figs. 1 and 2, Table 2) or long bones (Table 3). When using calvarial bones, isolate them and obtain calvarial disks as indicated above (Figs. 1 and 2). A set of calvarial disk remains intact, providing a baseline for gene expression, and another set of bones undergoes a series of collagenase‐EDTA (Ethylenediaminetetraacetic acid) digestions as indicated in Table 2. If using femurs and tibias, dissect them as indicated in the ex vivo Whole‐Bone Organ Cultures subsection. Cut off epiphyses and flush out the bone marrow. Once bones from all animals are collected, cut each bone in two pieces, and wash the bones with 1 mL of Hank's balanced salt solution (HBSS). Bones coming from one leg should be maintained in the incubator (no digested), and the ones coming from the other leg undergo a series of digestions as indicated in Table 3. Consistent with an enrichment in osteocytes, digested bones from both protocols should exhibit an increase in markers of osteocytes (ie, Sost, Dmp1), and a decrease in markers of osteoblasts (ie, Alpl, keratocan), compared with undigested bones. We recommend that the ratio between osteocytic and osteoblastic gene expression in intact bone versus osteocyte‐enriched bone be reported to demonstrate osteocyte enrichment. A sample size of at least five independent bone explants per condition should be used. Experiments should be repeated at least twice to confirm reproducibility of findings. Analysis of gene expression (RNA, Western blot, and ELISA), histology, μCT scanning, transcriptomic and proteomic approaches, and cellular and molecular signaling studies can be successfully performed with this protocol.

**Table 2:** Step‐by‐Step Protocol to Establish ex vivo Bone Organ Cultures Enriched in Osteocytes From Neonatal Calvarial Bones

Steps		Tips
1.	Dissect the calvarial bones, clean muscle and blood, and transfer to a 24‐well plate filled with sterile cold PBS.	Ideally, osteocyte‐enriched bone organ cultures are established with calvarial disks obtained from 7‐ to 9‐day‐old mice.
2.	Punch 2 holes in each calvaria using a 5‐mm biopsy punch, avoiding cranial sutures.	This step normalizes the amount of bone in each well/treatment condition, thus allowing the study of protein levels in conditioned media.
3.	Prepare a digestion solution: collagenase P (1.5 U/mL), trypsin (0.05%), and EDTA (1mM) in sterile PBS.	Prepare the mix fresh, before performing the digestions, and keep at 37°C.
4.	Incubate calvarial disks in 10 mL of digestion solution using a shaker at 37°C at 150 rpm.	We recommend not using more than 30 calvarial disks to enable a homogeneous digestion of the disks.
5.	After 20 min, remove the digestion solution and add 10 mL of fresh digestion solution. Repeat this step 10 times.	Digestion can be either discarded or kept for the isolation and culture of primary bone cells.
6.	Transfer digested calvaria disks to a 96‐well plate containing 200 μL of media per well.	Use αMEM media supplemented with 10% FBS and P/S.
7.	Collect conditioned media for assessing secreted protein levels and calvarial disks for mRNA expression.	Culture disks for up to 72 hours to achieve detectable levels of secreted factors in the conditioned media.

This protocol can be used with calvarias isolated from WT and genetically modified mice. When testing treatments in ex vivo bone organ cultures, we recommend performing dose‐ and time‐course experiments to determine the ideal conditions for your genes/proteins of interest.

αMEM = Minimum essential medium; P/S = Penicillin/Streptomycin; FBS = Fetal bovine Serum; EDTA = Ethylenediaminetetraacetic acid.

**Table 3:** Step‐by‐Step Protocol to Establish ex vivo Bone Organ Cultures Enriched in Osteocytes From Neonatal Long Bones

Steps		Tips
1.	Dissect the long bones (tibia and femurs), clean muscle, and blood.	Ideally, osteocyte‐enriched bone organ cultures are established with long bones obtained from 7‐ to 9‐day‐old mice.
2.	Remove soft tissue and periosteum via scraping and extensive washing, cut off epiphyses, flush out bone marrow, and cut each bone into 2 pieces. Place bone pieces in 1 mL of HBSS in 24‐well plates.	This step increases the surface of bone in contact with the digestion solution and improves the efficiency of the digestions.
3.	Prepare the digestion solutions: collagenase solution (300 active units/mL of collagenase type IA dissolved in αMEM with 1% of BSA), and EDTA solution (5mM, pH 7.4 prepared in DPBS with 1% of BSA).	Prepare the mix fresh, before performing the digestions, and keep at 37°C.
4.	Place bones from each animal into new wells containing 1.5 mL of collagenase solution. Incubate for 25 min on a rotating shaker set to 200 rpm in a 37°C and 5% CO_2_ humidified incubator.	Do not mix tibias and femurs; place in separate wells.
5.	After incubation, collect and discard digestion solution, and wash the bones once with HBSS. Repeat steps 4 to 5 two times more.	Cells obtained after digestions #3 to 5 can be kept and pooled together, and culture as the osteoblastic fraction.
6.	Incubate bone pieces with 1.5 mL of EDTA solution. Incubate for 25 min on a rotating shaker set to 200 rpm in a 37°C and 5% CO_2_ humidified incubator.	Cells obtained after digestions #7 to 9 can be kept and pooled together, and culture as the osteocytic fraction.
7.	After incubation, collect and discard digestion solution, and wash the bones once with HBSS.	
8.	Alternate collagenase digestions (steps 4 to 5) with EDTA digestions (steps 6 to 7) up to a total of 9 digestions.	
9.	After the final incubation, collect and discard digestion solution, and wash the bones once with HBSS.	
10.	Transfer digested bones to a 96‐well plate containing 200 μL of media per well.	Use αMEM supplemented with 10% FBS and P/S.
11.	Collect conditioned media for assessing secreted protein levels and bones for mRNA expression.	Culture disks for up to 72 hours to achieve detectable levels of secreted factors in the conditioned media. If measuring factors in the conditioned media, corrected by the weight of the bone fragments in each well.

This protocol can be used with long bones isolated from wild type and genetically modified mice. When testing treatments in ex vivo bone organ cultures, we recommend performing dose‐ and time‐course experiments to determine the ideal conditions for your genes/proteins of interest.

αMEM = Minimum essential medium; DPBS = Dulbecco's phosphate‐buffered saline; HBSS = Hank's balanced salt solution; P/S = Penicillin/Streptomycin; FBS = Fetal Bovine Serum; EDTA = Ethylenediaminetetraacetic acid; BSA = Bovine serum albumin.

## Use of ex vivo Bone Organ Cultures for the Study of Bone Biology

In this section, we will review some of the most recent findings obtained using ex vivo bone organ cultures and, when possible, compare the results obtained in these models with those seen in in vivo studies.

### Studies of osteocyte function

Ex vivo bone organ cultures have been used extensively to better understand the functions of osteocytes in bone. Using this approach, it was demonstrated that direct effects on osteocytes mediate some of the actions of Parathyroid hormone (PTH) on the skeleton.^21^, ^22^, ^23^, ^24^, ^25^, ^26^ For instance, the pro‐resorptive actions of PTH are associated with upregulation of the pro‐osteoclastogenic cytokine Rankl.^27^, ^28^, ^29^ Using genetic approaches, our group found in vivo that PTH signaling in osteocytes increases Rankl expression in bone through the distal control region (DCR) of the Rankl promoter.^21^ Likewise, treatment with PTH increased Rankl expression in ex vivo calvarial organ cultures established from WT mice. In contrast, PTH treatment did not increase Rankl in ex vivo organ cultures of bones from mice with genetic deletion of DCR, suggesting an important role of DCR in the upregulation of Rankl by PTH.^21^ To discern between effects of PTH on osteoblasts versus osteocytes, we employed ex vivo osteocyte‐enriched organ cultures. PTH treatment also increased Rankl expression in ex vivo osteocyte‐enriched organ cultures from tibias from WT but not from DCR^−/−^ mice, demonstrating that PTH upregulates Rankl expression in osteocytes through the DCR.^21^

Besides this direct effect of PTH on Rankl expression on osteocytes, using ex vivo organ cultures we described an additional mechanism by which PTH receptor signaling regulates Rankl‐mediated osteoclastogenesis. In vivo, genetic activation of PTH receptor 1 (Pth1r) signaling in osteocytes, as well as intermittent and chronic PTH elevation, increased in bone the expression of Mmp14, a transmembrane matrix metalloproteinase that generates soluble(s) Rankl after proteolytic cleavage of membrane‐bound Rankl.^23^ Similarly, treatment with PTH increased Mmp14 mRNA expression in calvarial bone organ cultures from WT mice. Importantly, a similar regulation of Mmp14 by PTH was observed in ex vivo organ cultures from calvarial bones enriched in osteocytes. However, Mmp14 upregulation by PTH was absent in osteocyte‐enriched bones from mice lacking the Pth1r in osteocytes.^23^ Together, these results demonstrated that Mmp14 is a target gene of the direct actions of PTH on osteocytes.

More recently, ex vivo osteocyte‐enriched organ cultures were used to study the expression and regulation of carbonic anhydrase III in osteocytes. It was found that carbonic anhydrase III is highly expressed in osteocytes; it is regulated by PTH, and protects osteocytes from oxidative stress.^26^

Osteocyte‐enriched ex vivo organ cultures have also been used to study the changes in gene expression patterns in osteocytes under pathologic conditions, as well as to identify differential gene expression between primary osteoblasts and osteocytes. In a recent article, Zimmerman and colleagues used ex vivo osteocyte‐enriched organ cultures to study the transcriptome of osteocytes in bones with osteogenesis imperfecta (OI) using RNA sequencing, and found that the osteocyte transcriptome is markedly dysregulated in OI, with important alterations in signaling pathways such as Wnt and Tgf‐β.^30^ Similarly, Stegen and colleagues employed ex vivo osteocyte‐enriched bone cultures to study the effects of oxygen in osteocytes and identified the oxygen‐sensing Phd2 as an important regulator of sclerostin and bone mass.^31^ Moreover, osteocyte‐enriched cultures have been used to study the effects of aging on osteocytic gene expression,^32^ the role of Piezo on osteocyte mechanotransduction,^33^ and the dysregulation of gene expression in osteocytes in X‐linked hypophosphatemia.^34^

Osteocyte‐enriched cultures have been also instrumental in determining the effects of conditional genetic deletion in osteocytes on the expression of target genes.^22^, ^33^, ^35^, ^36^, ^37^ This approach reduces the noise of other cells present in bone and allows a more accurate determination of the deletion of the gene of interest both at the DNA and RNA level. Finally, the expression of osteocyte‐secreted factors can and has been determined at the protein level in osteocyte‐enriched bone cultures, either in the conditioned media or in bone protein lysates.^23^ Additionally, the effects of osteocyte‐derived factors contained in conditioned media can be tested on other cells as reported earlier^38^ and discussed below in the Conclusions and Future Directions section.

Altogether, the examples provided in this section support that osteocyte‐enriched bone organ cultures are a reliable model to study osteocytes in their natural 3D bone microenvironment.

### Responses to hormones, therapeutic agents, and mechanistic studies

Besides providing a reliable approach to study bone cell interactions, ex vivo bone organ cultures can be also employed to study molecular signaling mechanisms in bone. We and others have demonstrated that whole‐bone organ cultures reproduce some of the typical molecular responses to PTH seen in vivo, including downregulation of Sost and upregulation of Rankl.^21^, ^22^, ^23^, ^24^, ^35^ As described above, we have also employed ex vivo organ cultures to identify a PTH‐induced new regulatory axis: Mmp 14‐sRankl.^21^, ^23^ In a recent example, ex vivo bone organ cultures were used to study the interplay between PTH signaling and Notch signaling in bone.^39^ Ex vivo cultures established with bones from WT mice and from genetically modified mice with Notch 2 gain‐of‐function allowed the investigators to evaluate the contribution of Notch receptor 2 signaling to the skeletal effects of PTH.^39^ Using a similar approach, Fulzele and colleagues employed ex vivo bone organ cultures using WT bones and bones with conditional deletion of the G‐protein α‐subunit in osteocytes to investigate the role of osteocytes in myelopoiesis.^38^

Ex vivo bone organ cultures have been extensively used to study the deleterious effects of glucocorticoids (GCs) on bone.^40^, ^41^ Similar to GC‐treated patients, we found that GCs decrease bone mass in WT mice, an effect accompanied by increased Sost/sclerostin expression in bone.^42^ In vivo, genetic deletion of Sost prevented GC‐induced bone resorption and loss of bone mass, suggesting that sclerostin mediates some of the damaging effects of GCs on bone.^42^ To further investigate the mechanisms behind these protective effects, we employed ex vivo organ cultures. We found that GCs increased Sost and decreased Opg expression, resulting in an increased Rankl/Opg ratio in ex vivo bone organ cultures from WT mice. Genetic deletion of Sost and treatment with an anti‐sclerostin neutralizing antibody in ex vivo bone organ cultures prevented OPG upregulation by GCs,^42^ supporting that blockade of Sost/sclerostin signaling mitigates the procatabolic effects of GCs on bone. More recently, we found that administration of GCs to mice in vivo upregulates in bone and muscle the expression of the proteasomal degradation inducers MuRF1, Atrogin1, and Musa1 (atrogenes), suggesting a potential role for these genes in GC‐induced weakness of bone and muscle.^43^ These findings were reproduced ex vivo in murine organ cultures of bone as well as skeletal muscle, providing the basis to search for pathways that potentially would interfere with GC action in both tissues. Following this rationale, we found that treatment with 1,25 vitamin D3 prevented GC‐induced atrogene upregulation in both bone and skeletal muscle ex vivo organ cultures.^44^ Inspired by these ex vivo findings, we perform an in vivo study to test the effects of 1,25 vitamin D3 in GC‐treated mice. Consistent with our ex vivo results, administration of 1,25 vitamin D3, or with another analog that also activates the vitamin D receptor, prevented atrogene upregulation induced by GC in bone and muscle and the decrease in bone mineral density (BMD) induced by GC, and partially preserved muscle function in vivo.^44^

We also used ex vivo bone organ cultures to perform mechanistic studies. We tested whether activation of β‐catenin in osteocytes increases Rankl via a sclerostin‐dependent mechanism.^45^ As seen in vivo, ex vivo cultures established bones from mice with constitutive activation of β‐catenin signaling in osteocytes (daßcat^Ot^) exhibited elevated sclerostin and Rankl mRNA expression, and treatment with anti‐sclerostin antibody reversed to control levels the increased expression of Rankl in bones from daßcat^Ot^ mice.^45^ Interestingly, the ex vivo organ culture model allows the study of the effects of genetic deletions without the need of crossing mice floxed for a particular gene with mice expressing the Cre recombinase, thus reducing costs and time. For instance, in a recent study, calvarial bones from miR21^fl/fl^ mice were treated with adenovirus‐Cre to delete the miR21 gene.^46^ Addition of adenovirus‐Cre decreased miR21 mRNA levels, an effect that, as seen in vivo, was accompanied by decreases in osteocyte viability.^46^ Finally, ex vivo bone organ cultures have been useful to investigate the bone effects of mechanical stimulation because of their ability to maintain the 3D distribution and canalicular network of osteocytes, the main mechanosensor cells in bone. Protocols and examples for the establishment of ex vivo bone organ cultures to study bone mechanobiology can be found in refs 47 to 49.^47^, ^48^, ^49^

In summary, the studies described here demonstrate that ex vivo bone organ cultures are a cost‐ and time‐effective approach to perform mechanistic studies, to evaluate the consequences of genetic deletions, to predict the effects of therapeutic interventions in bone, and to study mechanobiology in a controlled environment that reflects the cellular diversity and molecular complexity of the bone niche.

### Effects of cancer cells in bone

Primary, metastatic, and hematologic cancers can have devastating effects on bone. In addition, the growth of cancer cells in bone results in serious morbidity, including fractures, pain, nerve compression, and hypercalcemia.^50^ Cancer cells reside in distinct bone/bone marrow microenvironments where they establish reciprocal interactions with host cells that fuel tumor progression and bone disease.^51^ In the recent years, the introduction of drugs targeting the tumor and its environment has significantly improved patient outcomes. Thus, research efforts focus now on finding aberrant signaling pathways in the tumor cells and cells of the host niche to identify new molecular targets. In vitro models do not entirely reproduce the cellular diversity and the complexity of interactions in the tumor niche. Ex vivo bone organ cultures were developed in the late 1990s to model these interactions between cancer cells and the bone niche.^52^, ^53^ These culture systems recapitulate the spatial dimension, cellular diversity, and molecular networks of the tumor microenvironment. Moreover, ex vivo bone organ cultures are a useful model to simultaneously examine the anticancer efficacy and bone effects of therapies before moving to in vivo experimentation. A step‐by‐step guide to establish ex vivo cancer cell–bone organ cultures developed by our group can be found in Table 4 and in ref 54^54^ (Figs. 1 and 2).

**Table 4:** Step‐by‐Step Protocol to Establish ex vivo Cancer–Bone Organ Cultures

Steps		Tips
1.	Day 1, dissect the calvarial bones, clean muscle and blood, and transfer to a 24‐well plate filled with sterile cold PBS.	Ex vivo bone organ cultures can be established with calvarial disks obtained from 6‐ to 9‐day‐old pups immunodeficient or immunocompetent female/male mice.
2.	Punch 2 holes in each calvaria using a 5‐mm biopsy punch, avoiding cranial sutures (Fig. 1).	
3.	Transfer 5‐mm calvaria disks, concave side‐up, to a 96‐well plate containing 200 μL of media per well. Culture overnight.	
4.	Day 2, prepare a cell suspension of 50,000 cancer cells per 50 μL.	We use 50 μL of the cell suspension to ensure cancer cells are in close contact with the bone tissue. Locate the concave side of the calvarial disks up to facilitate retention of cancer cells.
5.	Remove media in the plate and add 50 μL (50,000 cancer cells) of the cell suspension slowly to the center of the calvarial disk and incubate at 37°C for 24 hours.	We recommend adding the cell suspension to the calvarial disk immediately after media removal to ensure disks do not dry out.
6.	Day 3, transfer calvarial disks with cancer cells to a new 96‐well plate.	This step is required to discard cancer cells that did not attach/engraft into the bone tissue and accumulate in edge of the wells without directly interacting with calvarial disks. We recommend using forceps placed close to the edge of the calvarial disks to transfer them to new plates.
7.	Fill wells with 200 μL of media and incubate at 37°C and 5% CO_2_. Refresh half of the media every 48 hours. Keep cultures up to 12 days.	
8.	Collect conditioned media for assessing secreted protein levels and calvarial disks for gene expression.	We recommend collecting conditioned media at different time points to assess the levels of secreted proteins and circulating markers (bone resorption, bone formation, tumor burden), and harvest calvarial disks for RNA/protein isolation and/or histology.

This protocol can be used with both human and murine cancer cell lines. When testing different cancer cells in ex vivo bone organ cultures, we recommend performing cell density and time‐course experiments to determine the ideal conditions for your endpoints of interest.

Using ex vivo cancer‐bone organ cultures, our group has examined the role of osteocytes in cancer in bone, particularly multiple myeloma, and determined the effects of several new therapeutic approaches on both cancer cells and bone cells. In our initial in vitro studies, we found that coculture with myeloma cancer cells increases the mRNA expression of Sost and activates Notch signaling in osteocytes.^55^ Correspondingly, we found that bones bearing myeloma cancer cells had increased mRNA expression of Sost and Notch target genes in ex vivo cancer–bone organ cultures.^55^ Importantly, these findings were reproduced in vivo, with bones infiltrated with myeloma cells displaying a higher percentage of osteocytes expressing sclerostin and activated Notch receptor 3 compared with control bones. Additionally, we recently described that in ex vivo cancer–bone organ cultures myeloma cells induce bone resorption and rapidly increase the levels of the circulating marker of bone resorption C‐telopeptide of type 1 collagen (CTX), and decrease the levels of the bone formation marker Type I procollagen N‐terminal propeptide (P1NP) in conditioned media collected,^56^ as occurs in animal models and patients.

Given the in vivo reproducibility of the results seen in ex vivo cancer–bone organ cultures, we have used this system to predict the effects of pharmacologic interventions. Aplidin, is a novel anti‐cancer compound isolated from the marine tunicate *Aplidium albicans* with antimyeloma activity in both in vitro and in vivo systems.^56^ Using ex vivo models, we investigated the effects of coadministration of aplidin with anti‐myeloma agents frequently used in the clinic on myeloma growth and bone.^56^ As seen in vivo, aplidin treatment decreased the number of tumor cells in our ex vivo cancer–bone model. Using the same model, we found that aplidin exhibits a potent antiresorptive activity, as determined by a dramatic decrease in the CTX levels found in the conditioned media. Co‐treatment with aplidin and dexamethasone or bortezomib decreased tumor burden further than each agent alone, enhancing aplidin's anti‐myeloma properties. Importantly, aplidin in combination with dexamethasone and bortezomib prevented the increased bone resorption induced by myeloma cells in ex vivo cancer–bone organ cultures. These findings provided a strong rationale to combine aplidin with other antimyeloma agents and to test the effects of these drug combinations in in vivo mouse models of myeloma. More recently, we characterized the effects of a novel bone‐targeted Notch inhibitor using our ex vivo cancer–bone organ cultures.^57^ We demonstrated that our bone‐targeted Notch inhibitor decreases the expression of Notch target genes and decreases tumor growth. Remarkably, similar results were observed in animal studies, with the bone‐targeted inhibitor decreasing specifically the expression of Notch target genes in bone, but not in other organs, and reducing tumor burden.^57^ Using a similar approach, Suvannasankha and colleagues reported that myeloma cells induce the expression of FGF23 in osteocytes, which in turn increases tumor growth and osteolysis.^58^

Ex vivo cancer–bone models are used for the study of other cancers that metastasize to bone. For instance, the effects of breast cancer cell lines on bone were tested in ex vivo cancer–bone organ cultures, using a combination of histologic and gene expression analyses.^59^, ^60^ Moreover, femur organ cultures have been employed to study breast cancer cell colonization of bone.^61^ Collectively, these results demonstrate that the ex vivo bone–cancer organ models can be a useful tool to study the effects of cancer cells in bone and to predict the effects of pharmacologic interventions in both bone and cancer cells.

### Ex vivo bone organ cultures as a bridge to human studies

Bridging the gap between animal research and human health has been challenging because of difficulties replicating findings in animal preclinical studies in humans. In an attempt to increase the reliability and clinical translation of findings in animal models, ex vivo bone organ cultures using human bone have been developed. Our first studies in this area aimed to determine the contribution of epigenetic DNA methylation marks to the regulation of the gene Sost in bone. In vitro work showed that treatment with a demethylating agent removed methyl marks from the Sost proximal promoter and stimulated sclerostin expression in human osteoblastic cell lines.^62^ To determine whether this could be a mechanism regulating Sost expression in human bone, we used ex vivo human bone organ cultures, and treated them with the same DNA methylation inhibitor. Similar to our in vitro studies, the expression of Sost in ex vivo bone organ cultures was increased.^62^ Clinical studies inspired by these initial findings demonstrated that Sost/sclerostin expression negatively correlates with DNA methylation at the Sost proximal promoter in several patient populations.^63^, ^64^ In a different set of experiments, we employed ex vivo human bone organ cultures to determine the translatability of findings found in mouse models. As described previously, PTH increases the expression of Mmp14 in murine bone, which in turn stimulates the production of sRankl.^23^ Consistent with these observations, PTH increased the expression of Mmp14 in bone and enhanced the release of sRankl to conditioned media in ex vivo human bone cultures. Remarkably, as occurred in our in vivo findings in mouse models, blockade of Mmp14 using an anti‐Mmp14 antibody decreased sRankl levels, demonstrating that the PTH–Mmp14–sRankl signaling axis is also active in human bone.^23^ Furthermore, human bone organ cultures have been used to examine the role of osteocytes in inflammatory bone loss^65^ and the expression pattern in osteocytes in osteoarthritic bone.^66^ Importantly, ex vivo cancer–bone organ cultures can also be performed with human subchondral bone disks.^67^ Although these models have several limitations (see Conclusion and Future Directions), ex vivo organ cultures using human bones can be used to confirm molecular mechanisms and to predict therapy responses, thus providing a cost‐effective bridge between basic science and translational medicine.

## Other ex vivo Organ Cultures

The skeleton is emerging as an important endocrine organ implicated in the regulation of whole‐body composition and glucose metabolism.^36^, ^68^, ^69^, ^70^ Similarly, bone and muscle communication has been a major focus of musculoskeletal research in the recent years.^71^, ^72^ Ex vivo muscle, pancreas, and fat organ cultures provide a valuable tool for not only exploring the mechanisms of gene regulation, cellular behaviors, and cell differentiation in these tissues, but also for the study of the cross‐talk between distant organs and the skeleton.

Ex vivo muscle organ cultures have been recently used by Brotto and collaborators to study the effects of osteocyte‐derived factors on muscle function; they found that conditioned media collected from MLO‐Y4 osteocyte‐like cells increased muscle contractile force by approximately 25% in ex vivo soleus organ cultures.^73^ As described above in the Responses to Hormones, Therapeutic Agents, and Mechanistic Studies subsection, similar to our observations of skeletal tissue in vivo, GCs increased the expression of atrogenes in ex vivo muscle cultures.^43^ Using this approach, we showed that GCs activate Notch signaling in muscle, which in turn elevate the expression of atrogenes, leading to muscle atrophy.

Our group recently started to use ex vivo fat and pancreas organ cultures to investigate the cross‐talk between bone and fat, and bone and pancreas. Growing evidence suggests that bone‐derived sclerostin regulates body composition and glucose metabolism.^36^, ^69^, ^70^, ^74^, ^75^, ^76^ We found that mice with elevated circulating sclerostin exhibit increased peripheral white fat mass and upregulation of genes involved in mitochondrial respiration and biogenesis including Pgc1α, Ucp1, or Prdm16. To determine if these changes in gene expression are caused by the direct effects of sclerostin on peripheral fat cells, we developed fat organ cultures and treated them with recombinant sclerostin.^77^ Consistent with the in vivo findings, treatment with sclerostin increased the expression of Pgc1α, Ucp1, and Prdm16 in ex vivo fat organ cultures.^77^ To test the effects of bone‐derived sclerostin on adipocyte differentiation, we established ex vivo bone organ cultures with bones from daßcat^Ot^, which overproduce sclerostin, and collected conditioned media. We found that treatment of adipocyte precursors with daßcat^Ot^‐derived conditioned media increase their differentiation,^77^ an effect fully blocked by treatment with an anti‐sclerostin antibody. In addition, high levels of sclerostin in serum were associated with impaired glucose tolerance, suggesting a potential role of bone‐derived sclerostin in the regulation of glucose metabolism. Treatment with recombinant sclerostin reduced the secretion of insulin in ex vivo organ cultures of pancreatic tissue.^77^ These findings suggest that sclerostin exerts endocrine actions in fat tissues and the pancreas to regulate body composition and glucose metabolism, respectively. Together, these results provide proof‐of‐concept of the potential of using organ cultures to study the cross‐talk between bone and muscle and bone and distant organs.

## Conclusions and Future Directions

In vitro and in vivo approaches are commonly used in the study of bone biology. In vitro cell cultures provide basic information on the function of individual bone cells and usually precede in vivo experimentation, which is ultimately considered the standard approach to determine the effects of genetic or pharmacologic interventions on bone. Ex vivo bone organ cultures serve as a transition model between in vitro and in vivo studies, as they reproduce the physical and spatial complexity of the bone microenvironment in a controlled setting (Figs. 1 and 2). Importantly, ex vivo bone organ cultures can be established with bones from multiples species, including humans, thus facilitating the translation of research findings into the clinic. Moreover, these cultures enable the study of osteocyte biology in a microenvironment that closely mimics the in vivo state. Further, ex vivo bone culture systems are easy to establish, cost‐efficient, easily manipulated, and have very few ethical limitations.

Ex vivo bone organ cultures are important tools to study several aspects of bone biology, including bone growth, bone and cartilage matrix turnover, responses to therapy, genetic manipulations, mechanical loading, the effects of cancer cells in bone, and the cross‐talk between bone and other organs. However, it is important to note that the traditional ex vivo bone organ cultures have several disadvantages, such as deficiency of blood supply, culture media perfusion, cell death and cell migration out of the bones after longer periods of culture, as well as the need to use highly rich culture media for applications requiring long‐term culture. Current research efforts are focused on reducing these limitations by incorporating bioreactors that increase tissue perfusion and the development of chorioallantoic membrane culture systems, as discussed in reference.^14^ In summary, ex vivo bone organ cultures are a unique and powerful platform for revealing cellular and molecular insights in bone biology, as well as the rapid screening of drugs and therapies.

## Disclosures

The authors declare no competing financial interests.
